# Bitter 2,5‐Diketopiperazines From Cocoa Activate Human Bitter Taste Receptors

**DOI:** 10.1002/mnfr.70618

**Published:** 2026-10-19

**Authors:** Silvia Schaefer, Adolfo Luna, Maik Behrens

**Affiliations:** ^1^ TUM Graduate School TUM School of Life Sciences Weihenstephan Technical University of Munich Freising Germany; ^2^ Leibniz Institute for Food Systems Biology at the Technical University of Munich Freising Germany

**Keywords:** 2,5‐diketopiperazine, bitter taste receptor, calcium‐mobilization assay, G protein‐coupled receptor

## Abstract

The human taste system enables a rapid analysis of nutritional and non‐nutritional food constituents. Although a bitter taste is considered a warning signal against ingesting potentially harmful substances, not all bitter substances are toxic, and a moderate bitterness is frequently tolerated or even preferred. Responsible for the detection of bitter substances in food are the ∼25 human bitter taste receptors residing in the oral cavity. An important class of bitter compounds found in thermally treated foods is the 2,5‐diketopiperazines, cyclic dipeptides made from two L‐amino acids, which are frequently formed as degradation products of polypeptides. Although 2,5‐diketopiperazines are established as major contributors to the bitter taste of many food items, it is still unknown which of the human bitter taste receptors mediate their detection. Using functional calcium‐mobilization assays, we identified two human bitter taste receptors, TAS2R14 and TAS2R43, being responsive to 2,5‐diketopiperazines. Of the two identified 2,5‐diketopiperazines activating TAS2R43, *cis*‐cyclo(L‐Leu‐L‐Phe) exhibited the highest potency with a threshold concentration of 100 µM. Further testing of 2,5‐diketopiperazine‐related linear dipeptides revealed that 2,5‐diketopiperazines represent their own class of bitter compounds, as no overlapping activation patterns with the corresponding dipeptides were evident.

## Graphical abstract

Thermal processing of protein‐containing food leads to the formation of cyclic dipeptides, called 2,5‐diketopiperazines (DKPs). These molecules often contribute to a bitter note in food. Despite the impact of DKPs on the taste of important food groups, their detection by human bitter taste receptors remained enigmatic. Using functional assays, two bitter taste receptors responsible for detecting DKPs were identified. The lack of activation of these receptors by the corresponding linear dipeptides suggests that DKPs represent a unique class of bitter compounds.

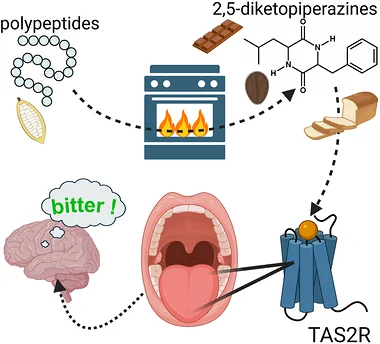

## 1 Introduction

With a market value of 106 billion US$ in 2017, chocolate is among the top commodities worldwide [1]. Being produced from seeds of the *Theobroma cacao* tree, cocoa, a key component of chocolate, became popular during the 18th century as an invigorating medicinal beverage [2]. Its sensory richness, including sweet, bitter, sour, astringent, and aromatic percepts, is a key characteristic of cocoa [3]. In particular, dark chocolate varieties are characterized by a well‐balanced bitterness arising from numerous compounds, which are believed to serve as protective agents for the cocoa plant against pathogens, insects, and herbivores [4, 5, 6]. Identified major contributors to the bitter taste of cocoa are alkaloids, such as caffeine and theobromine, as well as 2,5‐diketopiperazines, which are formed during roasting [4].

Bitter taste perception occurs in the oral cavity via the activation of bitter taste receptors, known as taste 2 receptors (TAS2Rs) [7, 8, 9]. The ∼25 putatively functional human TAS2Rs deviate in their bitter substance detection profiles considerably, with broadly tuned receptors being activated by numerous compounds and narrowly tuned receptors responding only to a few bitter agonists [10]. Combined, their activities cover a broad range of more than a thousand [11] chemically diverse bitter substances, both synthetic and natural. Despite being named “taste receptors”, the occurrence of bitter taste receptors is not limited to the oral cavity; they are found in numerous additional tissues, where they fulfill important physiological roles (for recent reviews see [12, 13]).

A number of key bitter tastants from cocoa have been assessed by sensory studies [14, 15] as well as by “unified flavor quantitation” [16]. These include the alkaloids caffeine and theobromine, several L‐amino acids, and cyclic dipeptides, the 2,5‐diketopiperazines, as well as a number of polyphenolic compounds characterized by inducing an astringent sensation in addition to their bitterness [14, 15]. Determination of compound concentrations in roasted cocoa nibs and the corresponding taste threshold concentrations revealed that not all compounds contribute equally to overall bitterness, as indicated by dose‐over‐threshold (DoT) factors of less than 1. The group of compounds that should contribute to the overall bitterness of cocoa includes the two alkaloids, several 2,5‐diketopiperazines, and polyphenols, whereas none of the bitter L‐amino acids reach their bitterness threshold concentrations. A considerable fraction of the identified cocoa bitter substances has been tested for their TAS2R activation profiles. Whereas caffeine was shown to activate five TAS2Rs, namely TAS2R7, ‐R10, ‐R14, ‐R43, and ‐R46 [10], theobromine only activated the single receptor TAS2R14 [17]. Also, the polyphenol (‐)‐epicatechin was demonstrated to activate the three TAS2Rs, TAS2R4, ‐R5, and ‐R39 [17, 18]. However, the strongly bitter 2,5‐diketopiperazines have never been tested for their activated receptor profiles until now.

2,5‐Diketopiperazines are cyclic dipeptides made from two L‐amino acids. They are small, rigid heterocyclic molecules and are found in a variety of organisms, including fungi, bacteria, plants, and animals. In food, they are frequently formed as degradation products of polypeptides upon processing, for example, heating [19]. Their known biological activities, apart from eliciting bitter taste, range from antimicrobial to anthroprotective effects [20].

To elucidate the contribution of 2,5‐diketopiperazines to the bitter taste in food in more detail, a functional screening of 26 human bitter taste receptors was performed. We selected six 2,5‐diketopiperazines identified in roasted cocoa nibs for the screening. To differentiate the bitterness elicited by 2,5‐diketopiperazines from the known bitter‐eliciting capacity of linear dipeptides [21, 22], we functionally tested the corresponding linear peptides as well. The results of this study have important implications for assessing the overall bitterness of complex food items, as modulatory effects arising from overlapping TAS2R activation profiles must be taken into account.

## 2 Experimental Section

### 2.1 Chemicals

The 2,5‐diketopiperazines *cis*‐cyclo(L‐Ala‐L‐Ala), *cis*‐cyclo(Gly‐L‐Phe), *cis*‐cyclo(L‐Leu‐L‐Pro), *cis*‐cyclo(L‐Leu‐L‐Phe), *cis*‐cyclo(L‐Phe‐L‐Pro), and cyclo(Gly‐L‐Pro) were purchased from Bachem (Weil am Rhein, Germany). For comparative analysis to cyclized forms, the linear peptides Gly‐L‐Phe, L‐Phe‐Gly, L‐Leu‐L‐Pro (L‐Leu‐L‐Pro‐HCl), L‐Pro‐L‐Leu, L‐Leu‐L‐Phe, Gly‐L‐Pro, and L‐Pro‐Gly were purchased from Bachem (Weil am Rhein, Germany), and L‐Phe‐L‐Leu was purchased from BroadPharm (San Diego, USA). All cyclic peptides were dissolved as stock solutions depending on their solubility in dimethyl sulfoxide (DMSO; Sigma–Aldrich, Cat #D2650). All linear peptides were dissolved as stock solutions in C1 buffer (130 mM NaCl, 5 mM KCl, 2 mM CaCl_2_, 10 mM HEPES, 10 mM glucose; pH 7.4). Peptide stock solutions were diluted to the desired maximum concentration with C1 buffer for functional calcium assays with a final DMSO concentration not exceeding 1%. All tested compounds and their stock solutions were stored at −20°C.

### 2.2 Bitter Taste Receptor Constructs

Existing expression constructs of the cDNAs of 26 human TAS2Rs [10, 23, 24] were present in pcDNA5/FRT (Invitrogen, San Diego, CA) or pEAK10 (Edge Biosystems, Gaithersburg, MD) plasmids, which included an aminoterminal sst3‐export tag and a carboxyterminal HSV‐tag.

### 2.3 Cultivation of Cells

HEK 293T cells stably expressing the chimeric G protein Gα16gust44 [25] were used for functional calcium mobilization assays as described before [26, 27]. Briefly, cells were grown in a monolayer in Dulbecco´s Modified Eagle Medium (DMEM) (Thermo Fisher Scientific, Cat# 41965‐02), supplemented with 10% heat‐inactivated fetal bovine serum (FBS) (Thermo Fisher Scientific, Cat# R‐7988438.2), 1% L‐glutamine (Sigma–Aldrich, Cat# G7513) and 1% penicillin–streptomycin solutions (Sigma–Aldrich, Cat# 15140‐122), in culture dishes coated with 1 µg/mL poly‐D‐lysine (Thermo Fisher Scientific, Cat# 17318583). The cells were incubated at cell culture conditions (37°C, 5% CO_2_ atmosphere, saturated air‐humidity).

### 2.4 Transient Transfections

For transient transfections, HEK 293T‐Gα16gust44 were seeded in 10 µg/mL poly‐D‐lysine‐coated clear‐bottom 96‐well plates (Greiner, Cat# 655090) to ∼30% confluence. The plates were maintained in cell culture conditions overnight in supplemented DMEM and allowed to reach 60%–70% confluence. Transfection was performed using 250 ng of plasmid DNA and 0.3 µL Lipofectamine 2000 (Invitrogen, Cat# 11668–500) per well in serum‐free DMEM. As a negative control, the empty pcDNA5/FRT vector (mock) was transfected. After incubation for 5 h at cell culture conditions, the medium was replaced with 100 µL of supplemented DMEM per well [26, 27].

### 2.5 Functional Calcium Mobilization Assays

Assays were performed ∼24 h after transfection as previously described [26, 27]. Briefly, cells were loaded with a solution of the calcium‐sensitive fluorescent dye Fluo‐4 AM (2 µM final concentration, Abcam, Cat# ab241082), in Dulbecco´s Modified Eagle Medium (DMEM) (Thermo Fisher Scientific, Cat# 41965‐02) in the presence of probenecid (2.5 mM final concentration, Sigma–Aldrich, Cat# P8761). Next, cells were incubated for 1 h in the dark at cell culture conditions, washed with C1 buffer using an automatic cell washer (BioTek 50 TS Microplate Washer, Merck KGaA, Darmstadt, Germany), and then incubated for 30 min in the dark at room temperature. Immediately before initiating the fluorescent calcium imaging assay with an automated fluorometric imaging plate reader (FLIPR^TETRA^, Molecular Devices, San Jose, USA), plates were washed again with C1 buffer.

After automated application of agonists, calcium responses were recorded at a wavelength of 510 nm following excitation at 488 nm. At the end of the measurement of test compounds, a cell viability control, somatostatin 14 (Bachem, Cat# 4033009) at a final concentration of 100 nM, was added to the cells. To calculate the relative change in fluorescence (Δ*F*/*F*) stimulated by each peptide solution, the fluorescence amplitude observed for receptor‐transfected cells was mock‐subtracted using the corresponding identically treated cells transfected with an empty vector and normalized to background fluorescence. The data from three independent experiments, each performed in duplicates, were checked for statistical significance using a two‐sided Student's *t*‐test (*p* ≤ 0.05).

### 2.6 Dose–Response Relationships and Statistics

Dose–response relationships were calculated for each TAS2R and each peptide agonist. For the calculation, the Δ*F*/*F* of mock‐transfected cells was subtracted from that of TAS2R‐transfected cells and normalized to the background fluorescence. Normalized fluorescence changes were averaged from the two wells and three independent experiments of each peptide concentration tested. A nonlinear regression analysis was performed to create a four‐parameter logistic curve and to calculate the EC_50_‐concentration using SigmaPlot 14.0 (Systat Software Inc.). The equation to fit the data was: *y* = min+(max‐min)/1+(x/EC_50_)^−Hillslope^. The threshold concentrations were defined as the lowest peptide concentration that significantly increased calcium signaling in receptor‐transfected cells compared to mock‐transfected cells (*p* ≤ 0.05).

## 3 Results

To identify human bitter taste receptors responding to 2,5‐diketopiperazines, we screened 26 putatively functional human TAS2Rs expressed in HEK 293T‐Gα16gust44 cells with *cis*‐cyclo(Gly‐L‐Phe), *cis*‐cyclo(L‐Leu‐L‐Pro), *cis*‐cyclo(L‐Leu‐L‐Phe), *cis*‐cyclo(Gly‐L‐Pro), *cis*‐cyclo(L‐Phe‐L‐Pro), and *cis*‐cyclo(L‐Ala‐L‐Ala) (Figure 1; Figures S1–S3).

**FIGURE 1:**
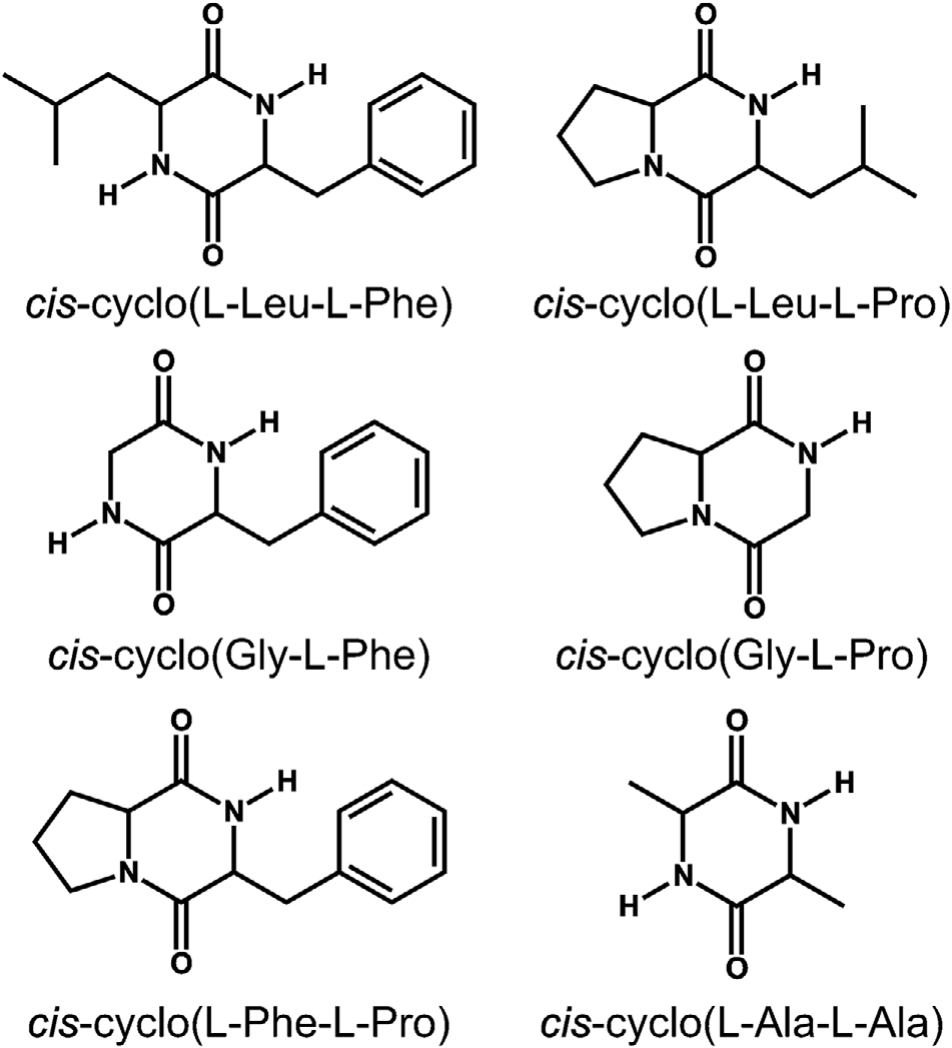
Structural formulas of six 2,5‐diketopiperazines previously identified in roasted cocoa nibs. The diketopiperazines were used for functional calcium mobilization assays to identify responding human bitter taste receptors.

Due to the limited water solubility of the 2,5‐diketopiperazines, stock solutions in DMSO were prepared and diluted into the C1‐buffer. Initial experiments revealed that receptor‐independent cellular artifacts as well as solubility issues limited the maximally applicable concentrations individually to: 80 mM for *cis*‐cyclo(L‐Ala‐L‐Ala), 3 mM for *cis*‐cyclo(Gly‐L‐Phe), *cis*‐cyclo(L‐Leu‐L‐Pro), *cis*‐cyclo(Gly‐L‐Pro), and *cis*‐cyclo(L‐Phe‐L‐Pro); and 0.15 mM for *cis*‐cyclo(L‐Leu‐L‐Phe). While most TAS2Rs and most 2,5‐diketopiperazines did not lead to specific activation of transfected cells, two receptors and two substances resulted in specific fluorescence signals (Figure 2). The TAS2R43 was selectively activated by the substance *cis*‐cyclo(L‐Leu‐L‐Phe), whereas the receptor TAS2R14 responded to *cis*‐cyclo(L‐Leu‐L‐Phe) as well as to *cis*‐cyclo(L‐Leu‐L‐Pro).

**FIGURE 2:**
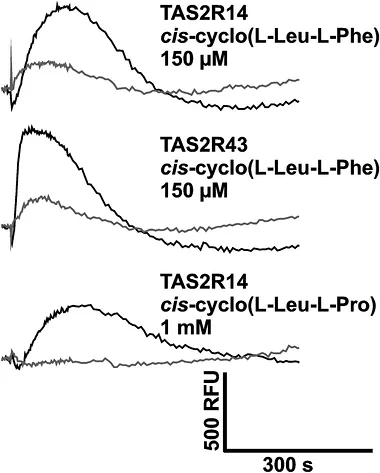
Raw fluorescence traces of TAS2Rs responding to 2,5‐diketopiperazines. The fluorescence traces of HEK 293T‐Gα16gust44 cells expressing TAS2R14 and TAS2R43 after stimulation with 2,5‐diketopiperazines are shown (black traces). Identically treated cells transfected with an empty expression vector (= mock control) served as negative controls (gray traces). The substance concentrations used are indicated. A scale bar showing relative fluorescence units (RFU, *y*‐axis) and time in seconds (s, *x*‐axis) is provided at the bottom.

To determine the dose–response relationships of the two activating 2,5‐diketopiperazines at the two bitter taste receptors, further experiments using increasing agonist concentrations were performed. For the 2,5‐diketopiperazine *cis*‐cyclo(L‐Leu‐L‐Phe) an EC_50_‐concentration of 0.041 ± 0.013 mM has been determined for activation of TAS2R43, whereas, due to a lack of signal saturation, an EC_50_‐concentration for this compound's activation of TAS2R14 could not be calculated. However, from the response curve shown in Figure 3, left panel, it is evident that *cis*‐cyclo(L‐Leu‐L‐Phe) exhibits higher potency and efficacy with TAS2R43 than with TAS2R14‐expressing cells. In contrast to *cis*‐cyclo(L‐Leu‐L‐Phe), *cis*‐cyclo(L‐Leu‐L‐Pro) activated only TAS2R14 with an EC_50_‐concentration of 0.269 ± 0.074 mM, albeit with reduced efficacy. For the threshold concentration, which was defined as the lowest agonist concentration leading to a statistically significant activation of receptor‐expressing cells compared to identically treated mock‐transfected cells, a rank order of potency for *cis*‐cyclo(L‐Leu‐L‐Phe) (0.1 mM for TAS2R43 and TAS2R14), being slightly more potent than *cis*‐cyclo(L‐Leu‐L‐Pro) (0.3 mM for TAS2R14), was observed.

**FIGURE 3:**
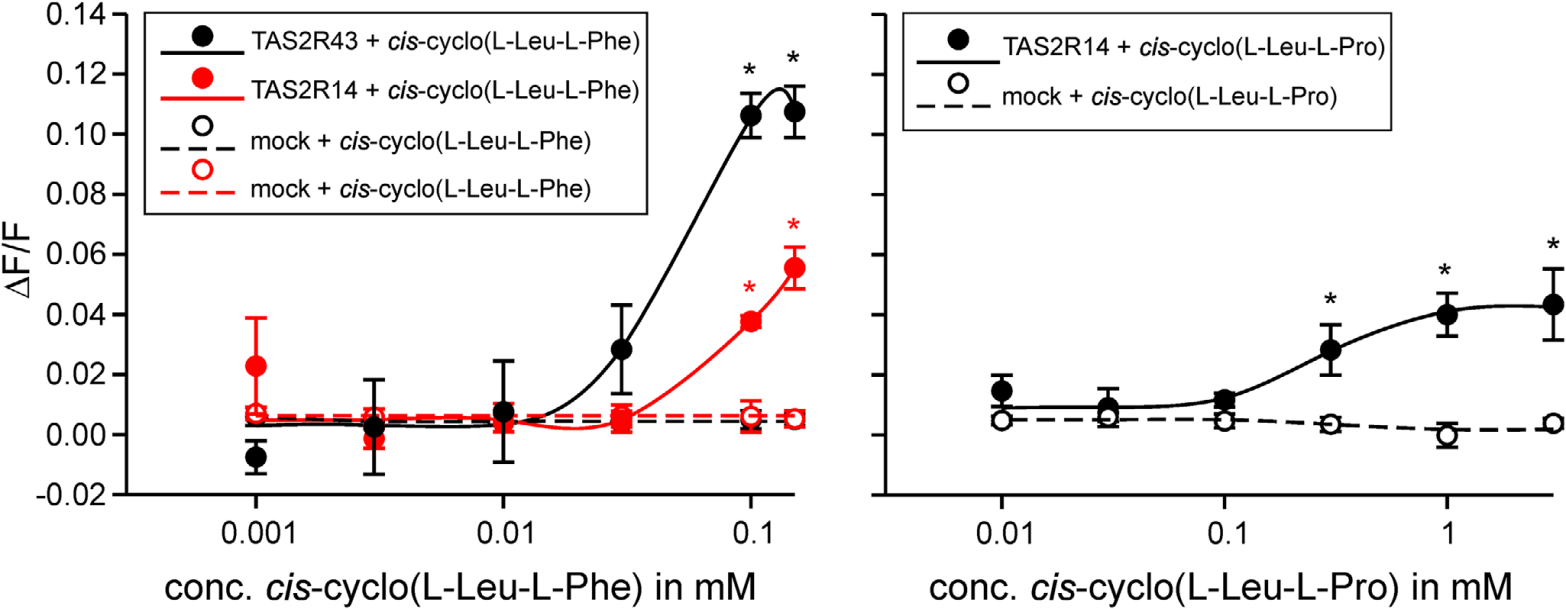
Dose–response relationships of 2,5‐diketopiperazines with TAS2R14‐ and TAS2R43‐expressing cells. HEK 293T‐Gα16gust44 cells transiently transfected with TAS2R43 and TAS2R14 were challenged with increasing concentrations of *cis*‐cyclo(L‐Leu‐L‐Phe) (left panel) and *cis*‐cyclo(L‐Leu‐L‐Pro) (right panel) using a fluorometric imaging plate reader (FLIPR^tetra^). After the automated substance application, the fluorescence emitted by the calcium‐sensitive fluorescent dye Fluo4‐am was monitored and used to calculate the relative changes in fluorescence (Δ*F*/*F*, *y*‐axis). The substance concentrations in mM are plotted on the logarithmically scaled *x*‐axis. Asterisks indicate statistically significant differences (*p* ≤ 0.05) between receptor‐expressing and identically treated mock‐transfected cells. The lowest concentrations leading to significant differences between receptor‐ and mock‐transfected cells were considered the threshold concentrations. Three independent experiments with two technical replicates each were performed.

The finding that 2,5‐diketopiperazines activate two human bitter taste receptors raised the question of whether the cyclic nature of the compounds is responsible for their bitterness or whether linear peptides could also elicit responses, as shown before [21, 22, 27, 28], or rather not [29]. Therefore, linear variants of the 2,5‐diketopiperazines, namely, Gly‐L‐Phe, L‐Phe‐Gly, L‐Leu‐L‐Pro, L‐Pro‐L‐Leu, L‐Leu‐L‐Phe, L‐Phe‐L‐Leu, Gly‐L‐Pro, and L‐Pro‐Gly, were functionally tested with all 26 TAS2Rs. Of the eight tested linear dipeptides, only 3 activated single receptors at rather high concentrations. Similar to the dipeptide L‐Leu‐L‐Pro, Gly‐L‐Pro activated the receptor TAS2R43. This activation pattern appears to be unrelated to the receptor's response to the 2,5‐diketopiperazine, *cis*‐cyclo(L‐Leu‐L‐Phe), which has another amino acid composition. The linear dipeptide L‐Leu‐L‐Phe elicited responses of TAS2R2‐expressing cells, a receptor that has not shown any activation by 2,5‐diketopiperazines in our screening experiments before (Figure 4; Figures S4–S7). Therefore, the bitter responses to 2,5‐diketopiperazines are apparently not related to general peptide detection.

**FIGURE 4:**
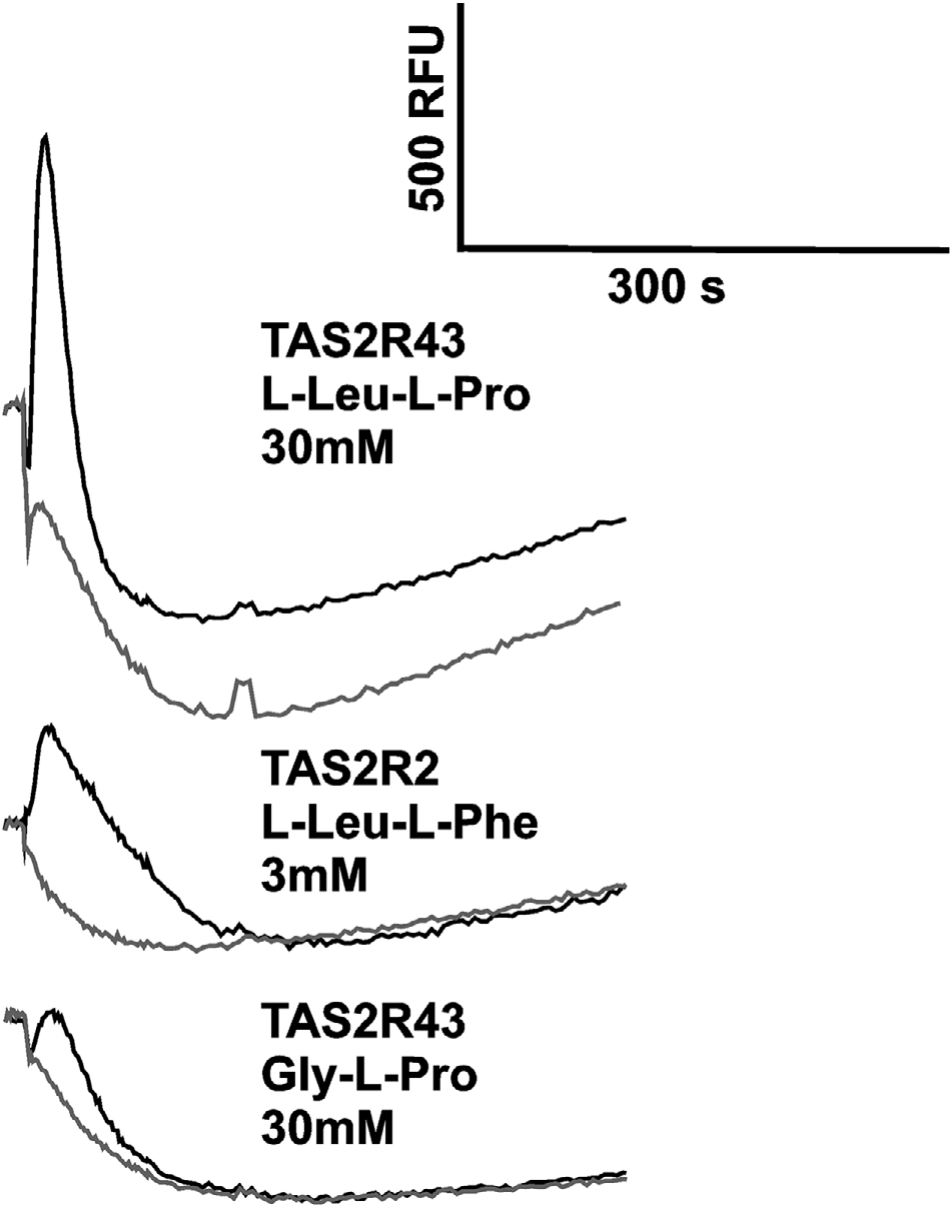
Raw fluorescence traces of 2,5‐diketopiperazine‐related linear dipeptides. The fluorescence traces of HEK 293T‐Gα16gust44 cells expressing TAS2R2 and TAS2R43 after stimulation with 2,5‐diketopiperazine‐related linear dipeptides are shown (black traces). Identically treated cells transfected with an empty expression vector (= mock control) served as negative controls (gray traces). The substance concentrations used are indicated. A scale bar showing relative fluorescence units (RFU, *y*‐axis) and time in seconds (s, *x*‐axis) is provided at the top.

## 4 Discussion

The present study investigated the bitter taste receptor‐activating properties of 2,5‐diketopiperazines, which are present in roasted cocoa nibs. Of the six 2,5‐diketopiperazines tested for the activation of human bitter taste receptors, only two elicited responses in vitro. According to Stark and Hofmann, the compound *cis*‐cyclo(L‐Leu‐L‐Phe) was among the 2,5‐diketopiperazines identified in roasted cocoa nibs, exhibiting with 190 µM the lowest bitter threshold of all sensorially tested 2,5‐diketopiperazines [30]. This is in good agreement with the receptor data presented in this study, which demonstrated an activation threshold of 100 µM for the two receptors TAS2R43 and TAS2R14. The other 2,5‐diketopiperazine that was shown to activate TAS2R14 in our study, the *cis*‐cyclo(L‐Leu‐L‐Pro), exhibited a lower potency with a bitter taste threshold of 1190 µM according to Stark and Hofmann [30], whereas the in vitro threshold reported in this study was 300 µM. Hence, the rank‐order of potency agrees well with regard to the two 2,5‐diketopiperazines found positive; the threshold concentration deviates somewhat. Another 2,5‐diketopiperazine, *cis*‐cyclo(L‐Ala‐L‐Ala), showed a bitter taste threshold of > 7000 µM in a sensory study [30], which agrees with the absence of responses in the in vitro study presented here. This is likely also true for the compound *cis*‐cyclo(Gly‐L‐Pro), which showed a sensory bitter threshold of 3250 µM [30] and did not show an activation of TAS2Rs at the maximum test concentration of 3000 µM. In contrast to the above data showing a good agreement between sensory tests and the in vitro data presented in this study, other negative findings remain unexplained. The bitter taste threshold concentrations of *cis*‐cyclo(Gly‐L‐Phe) with 610 µM and of *cis*‐cyclo(L‐Phe‐L‐Pro) with 1020 µM, as determined by previous sensory experiments [30], were within the concentration range used in the in vitro screening of TAS2Rs. Whether a bitter compound can be tasted in food depends on its actual concentration, which should be higher than the sensory threshold concentration. The DoT factors for the 2,5‐diketopiperazines used in this study were also determined for roasted cocoa nibs by Stark and Hofmann and ranged between 0.6 and < 0.1. Therefore, a direct contribution to the bitterness of cocoa nibs investigated in the latter study cannot be concluded. However, the overall content of 2,5‐diketopiperazines in chocolates made with different cocoa seed varieties fluctuates substantially and may even serve as potential markers for the used beans [31].

Moreover, for complex food items, additional factors need to be taken into account, including the possibility for additive effects, for example, several compounds at subthreshold targeting the same receptor and reaching the activity‐inducing concentration, or, on the contrary, agonistic and antagonistic activities compete for the same receptor, leading to reduced activation [32]. Roasted cocoa nibs and products thereof are by far not the only food items that contain 2,5‐diketopiperazines in different compositions and concentrations. In fact, since they occur through thermal food processing from practically ubiquitous peptide constituents [19], 2,5‐diketopiperazines have been detected, in addition to cocoa, in roasted malt (beer) [33, 34], coffee [35, 36], chicken broth [37], and also in aged sake [38] and comté cheese [38]. Particularly high concentrations of 2,5‐diketopiperazines can be reached in bread upon baking. The study by Ryan and colleagues reported for cyclo(L‐Leu‐L‐Pro) concentrations of up to 336 µg/kg in bread crumb and up to 23.0 mg/kg in bread crust, respectively [39], which is within the activating concentration range of the receptor TAS2R14 (Figure 3, right panel) and clearly exceeds the threshold concentration for this compound as determined by Stark and Hofmann [30].

Concerning the bitter taste receptors activated by the 2,5‐diketopiperazines in this study, it is not surprising that one of them is the TAS2R14. This receptor has been the first of the human TAS2Rs identified as broadly tuned [40], a finding confirmed and expanded by numerous studies (e.g., [10, 26, 41, 42, 43, 44, 45]) resulting in the impressive number of about 150 chemically diverse cognate agonists [11]. The receptor responding with the highest sensitivity to *cis*‐cyclo(L‐Leu‐L‐Phe) was the TAS2R43 (Figure 3). Previous research identified the activation of this receptor by various bitter compounds in food and beverages, such as convicine from faba beans [46], mozambioside and related compounds from coffee [32, 47, 48], several sesquiterpene lactones from chicory [49], cyclolinopeptides from linseed oil [29], and the synthetic sweeteners saccharin and acesulfame K [50, 51], suggesting a prominent role of TAS2R43 for the bitter taste perception of our diets. Somewhat surprising were the responses of TAS2R43 and TAS2R2 [23, 52] to linear peptides, as these do not belong to the 5 TAS2Rs biased to bitter peptide detection [21, 27]. Whereas the just recently characterized TAS2R2 may have been overlooked as a peptide‐responsive receptor previously, the TAS2R43 may have detected a pharmacophore not previously tested.

In conclusion, our experiments with the 2,5‐diketopiperazine‐related linear dipeptides suggest that 2,5‐diketopiperazines represent a unique class of bitter substances rather than another form of dipeptides, as we have not observed an overlapping activation pattern of cyclic and linear peptides for any of the TAS2Rs. The conserved central structure of 2,5‐diketopiperazines, their variability, and their occurrence in numerous food items indicate the possibility for substantial competitive effects at the level of TAS2Rs, a topic that should be investigated in the future.

## Author Contributions

**Silvia Schaefer**: writing – original draft preparation, writing – review and editing, and supervision. **Adolfo Luna**: writing – original draft preparation, writing – review and editing, and visualization. **Maik Behrens**: conceptualization, writing – original draft preparation, writing – review and editing, visualization, and supervision. All authors have read and agreed to the final version of the manuscript.

## Funding

This research was supported by the Deutsche Forschungsgemeinschaft (DFG − German Research Foundation) (BE 2091/7‐1 to MB).

## Conflicts of Interest

The authors declare no conflicts of interest.

## Supporting information




**Supporting File**: mnfr70618‐sup‐0001‐SuppMat.pdf.

## Data Availability

The data that support the findings of this study are available from the corresponding author upon reasonable request.
