# Supplementary material for: Bitter 2,5‐Diketopiperazines From Cocoa Activate Human Bitter Taste Receptors
Source: Mol Nutr Food Res. 2026 Oct 19;70(20):e70618. doi: 10.1002/mnfr.70618 (PMC13678406; doi:10.1002/mnfr.70618)

Supporting information

The following supplementary figures summarize exemplary raw fluorescence traces obtained during the screening of the 26 TAS2Rs with six 2,5-diketopiperazines and eight derived linear dipeptides. The compounds and their final applied concentrations are indicated at the top of each panel and were selected based on pilot experiments yielding tolerable/no receptor-independent artifact signals. The fluorescence traces shown in red lines cover the entire measurement period of ~804 seconds and are automatically scaled (software: Screenworks 4.2). The positive signals developing after the application of 2,5-diketopiperazin or a dipeptide at ~19 seconds are highlighted with green arrows. Ambiguous traces were shown together with negative control traces (black) to demonstrate the lack of convincing receptor-specific signals. A second application of 100 nM somatostatin-14 (Bachem, Cat# 4033009) at ~360 seconds served as viability control, as evidenced by the rapid increase in fluorescence due to endogenous somatostatin receptor signaling. The blue traces originate from TAS2R14-expressing cells stimulated with 10 µM aristolochic acid, serving as a positive control.

List of supporting figures

Supporting figure S1	*cis*-cyclo(L-Leu-L-Pro), *cis*-cyclo(L-Leu-L-Phe)	page 2
Supporting figure S2	*cis*-cyclo(Gly-L-Phe), *cis*-cyclo(L-Phe-L-Pro)	page 3
Supporting figure S3	*cis*-cyclo(Gly-L-Pro), *cis*-cyclo(L-Ala-L-Ala)	page 4
Supporting figure S4	L-Phe-L-Leu, L-Leu-L-Phe	page 5
Supporting figure S5	L-Leu-L-Pro, L-Pro-L-Leu	page 6
Supporting figure S6	Gly-L-Pro, L-Pro-Gly	page 7
Supporting figure S7	L-Phe-Gly, Gly-L-Phe	page 8

Supporting figure S1

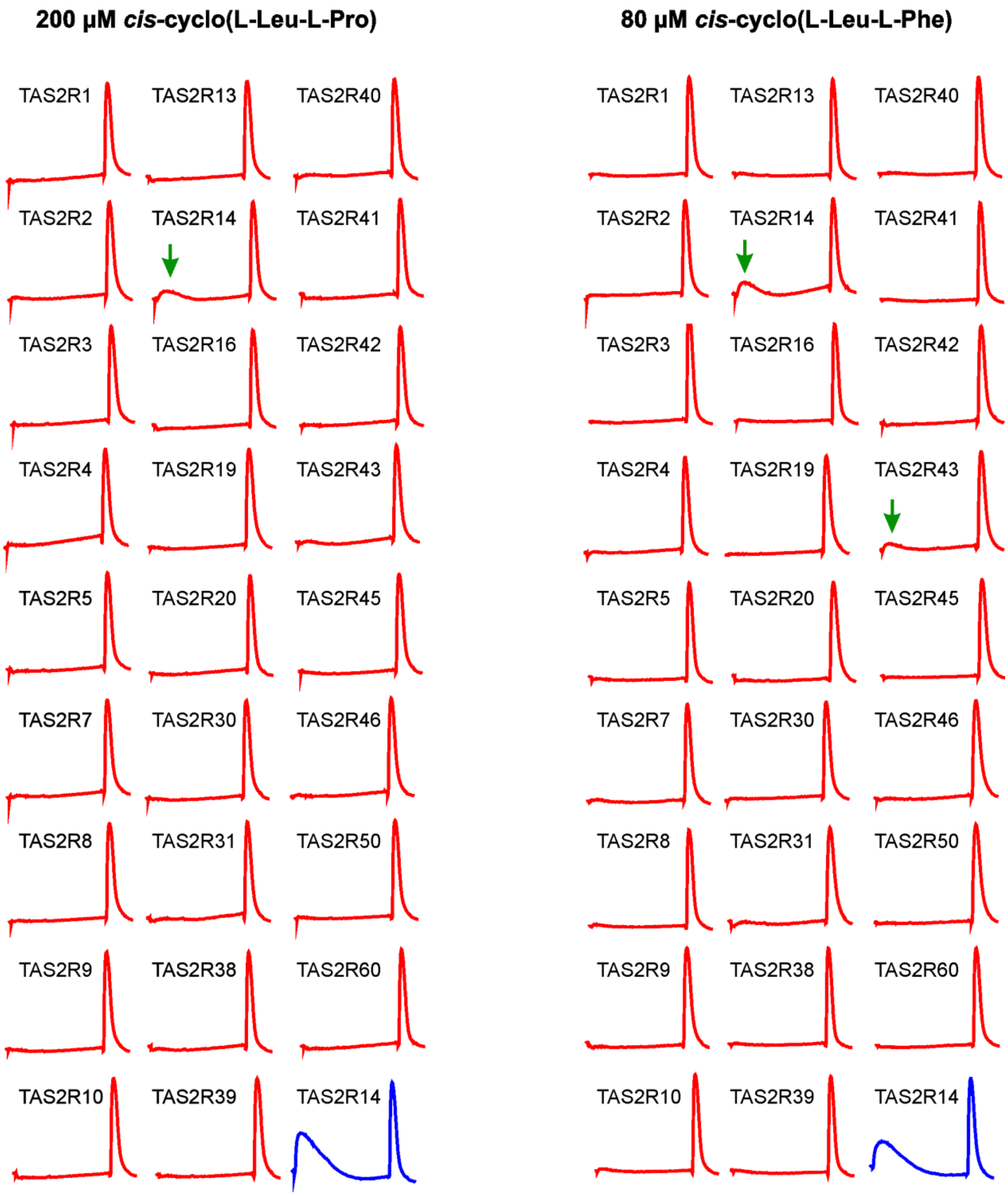

Supporting figure S2

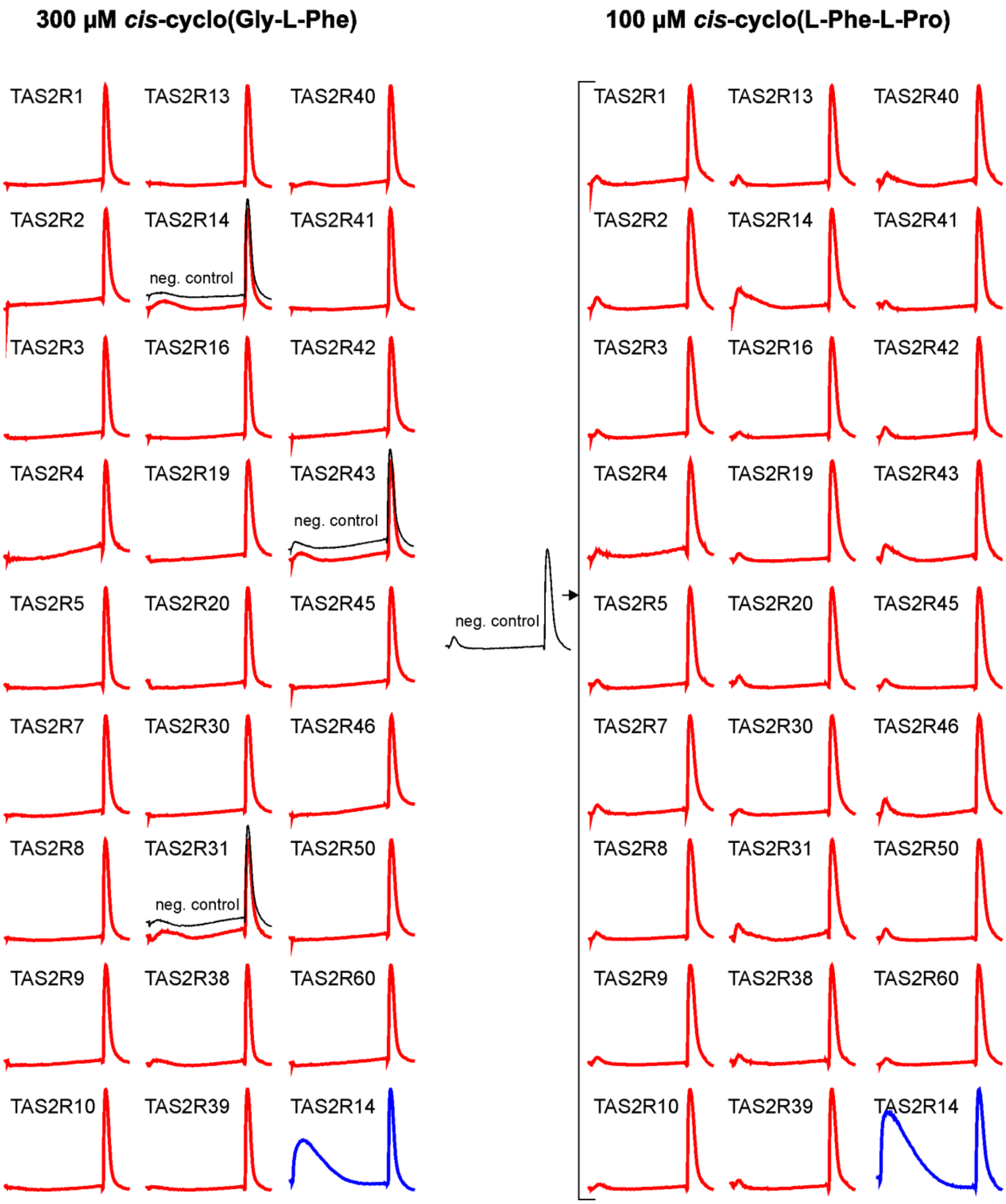

Supporting figure S3

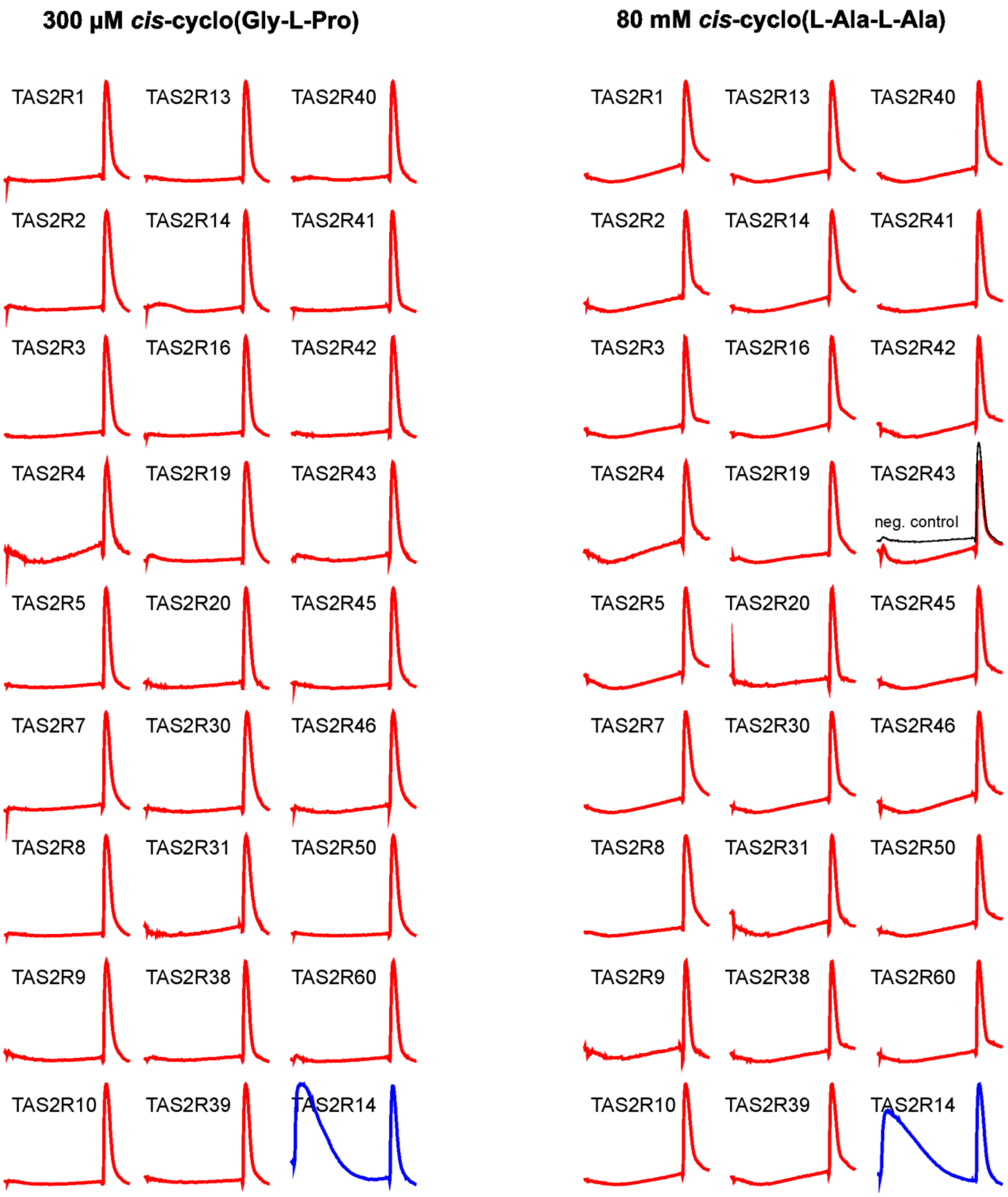

Supporting figure S4

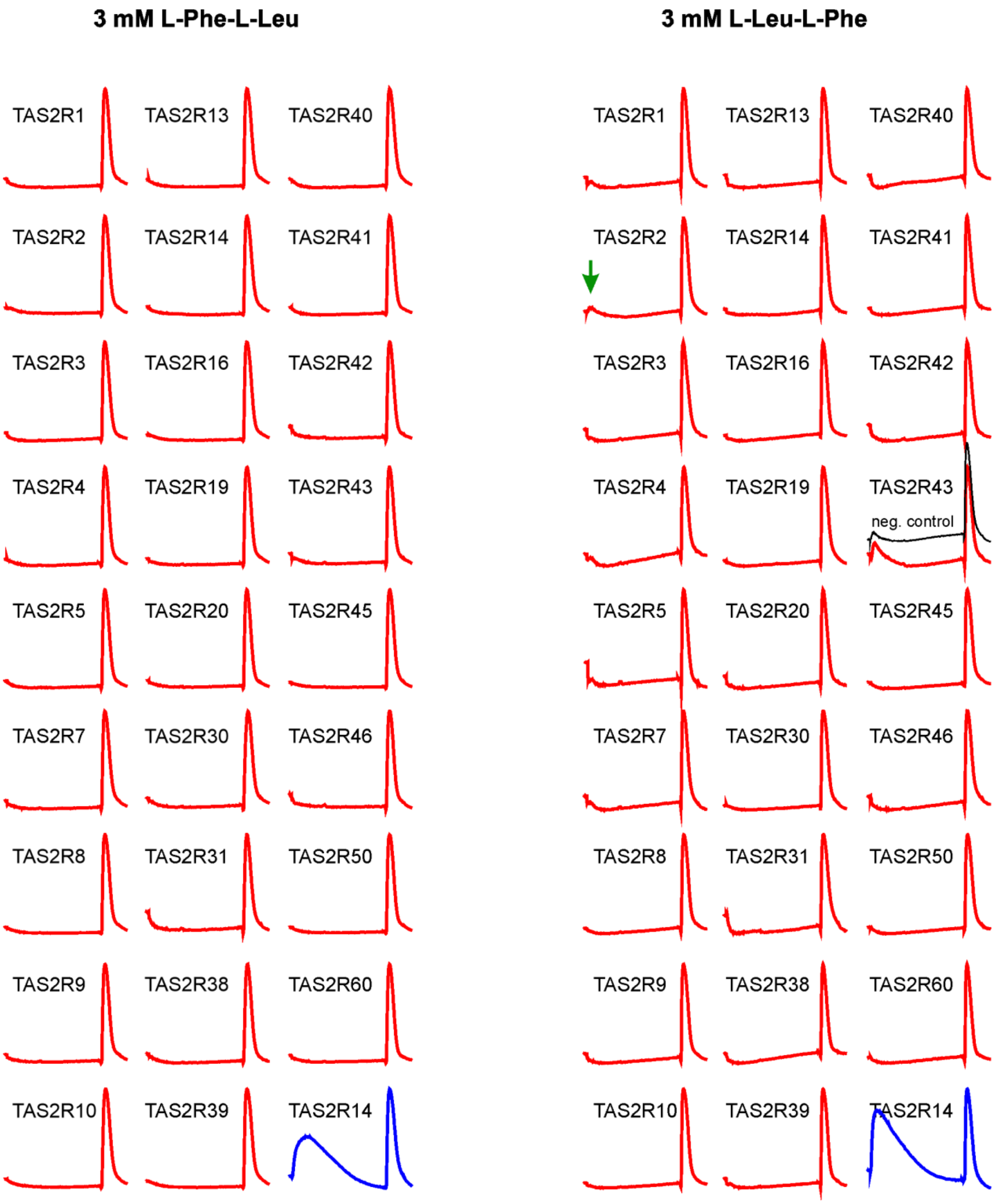

Supporting figure S5

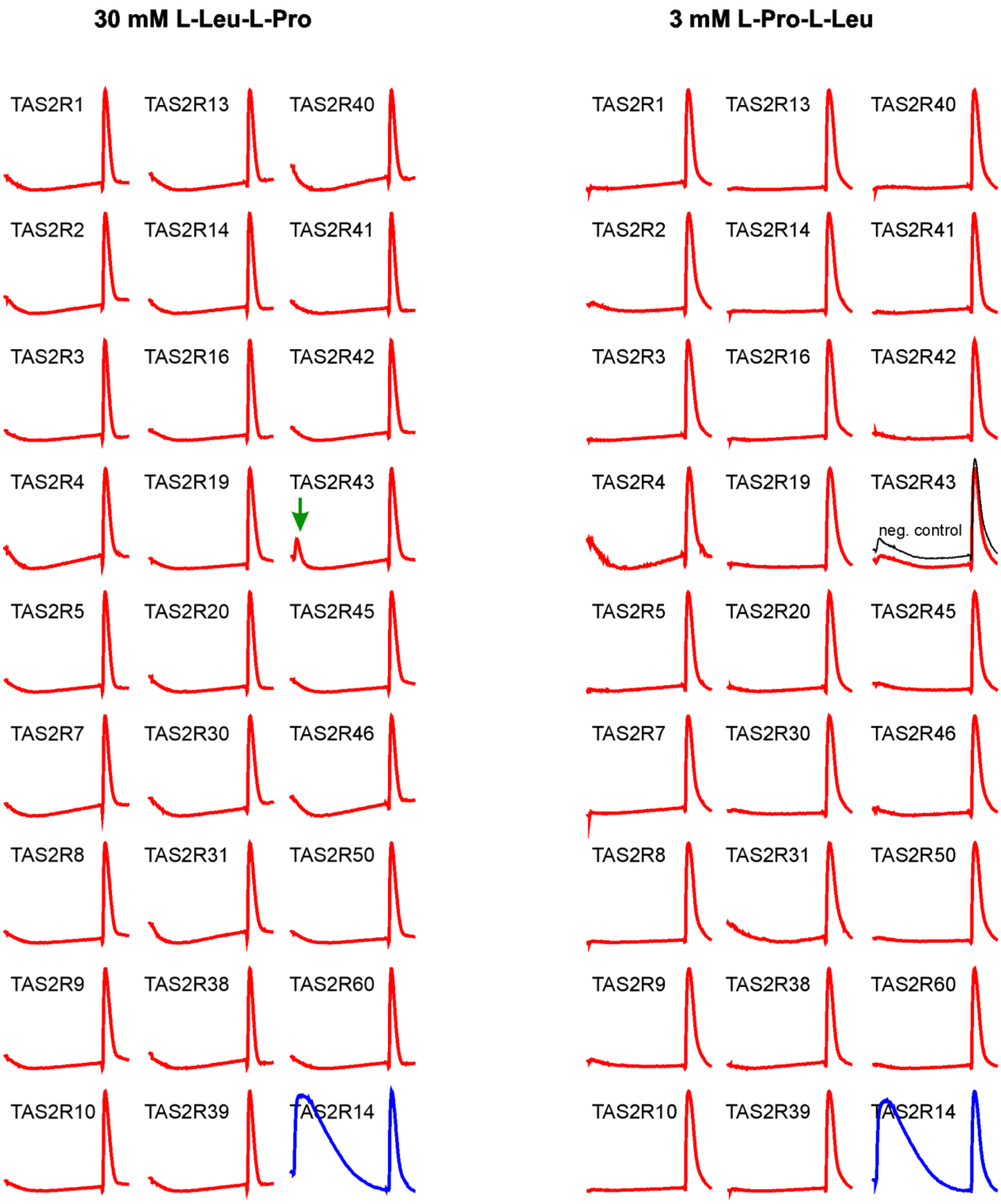

Supporting figure S6

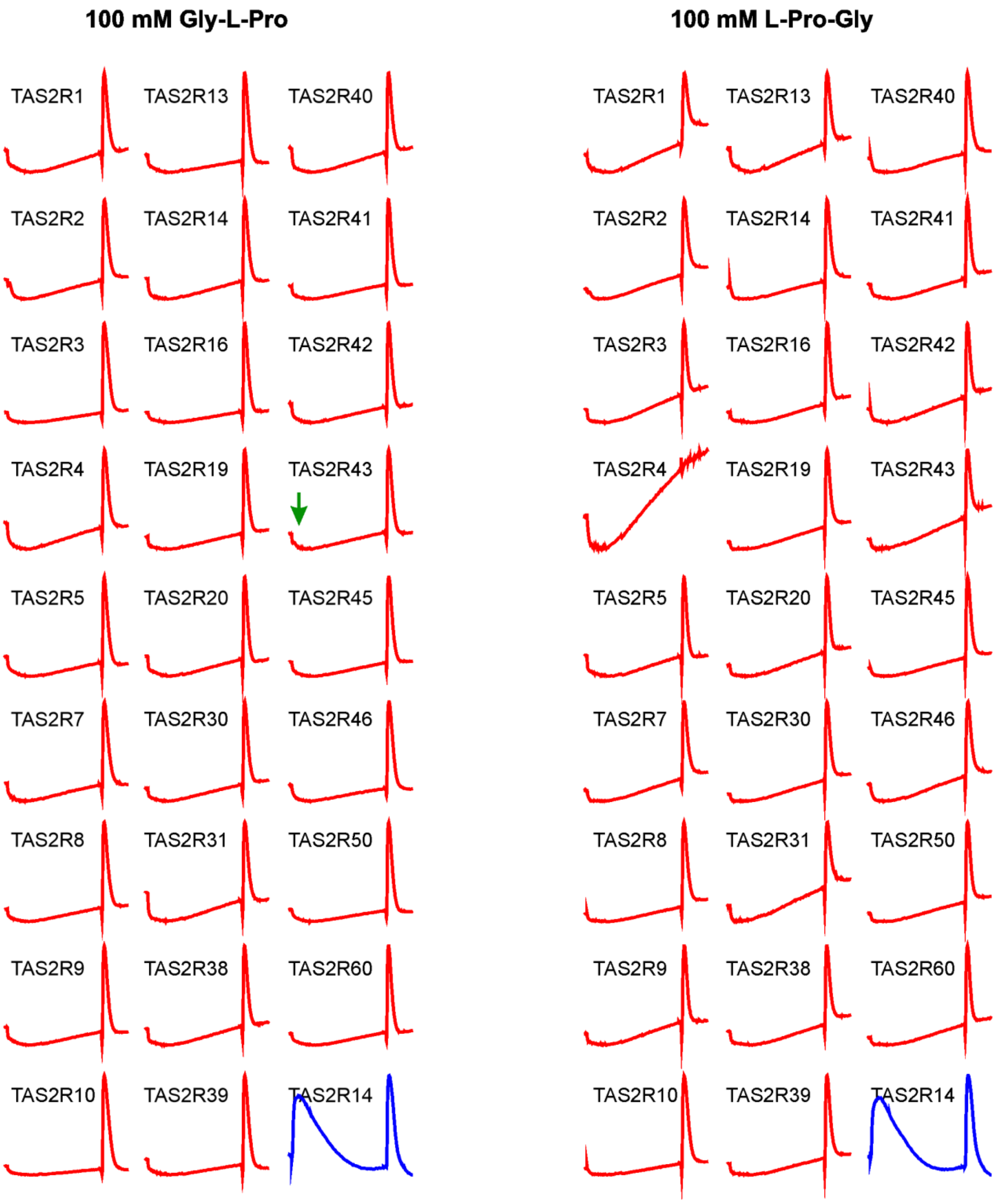

Supporting figure S7

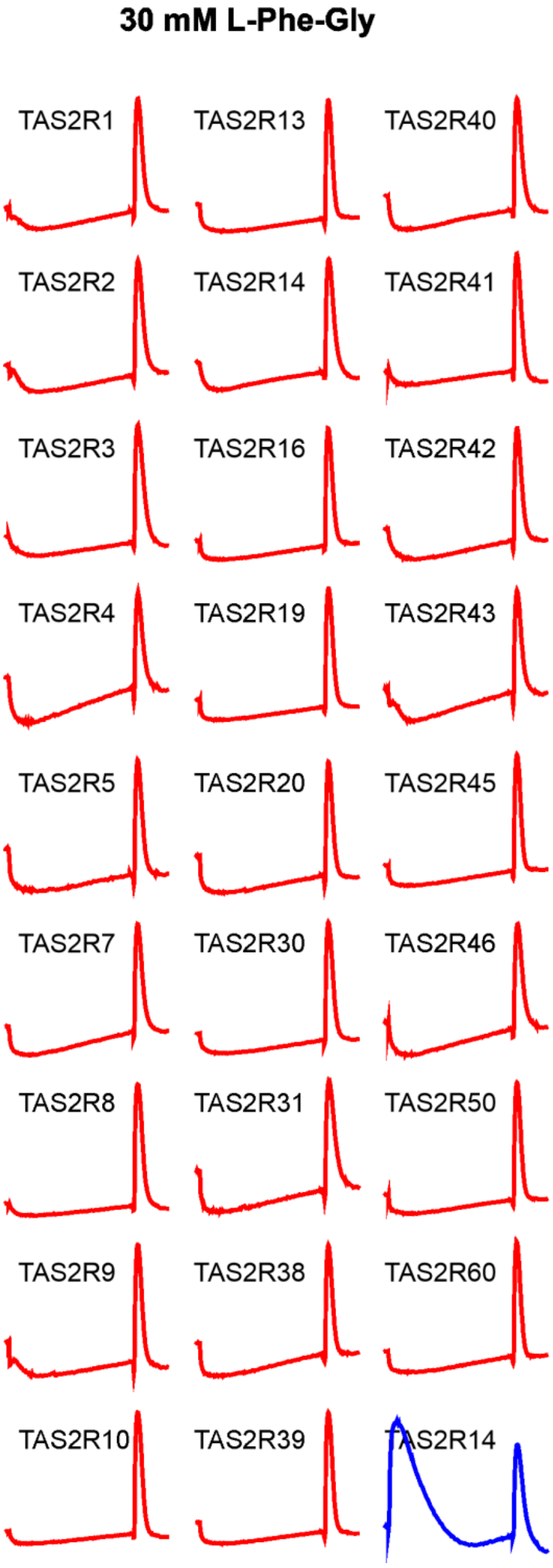

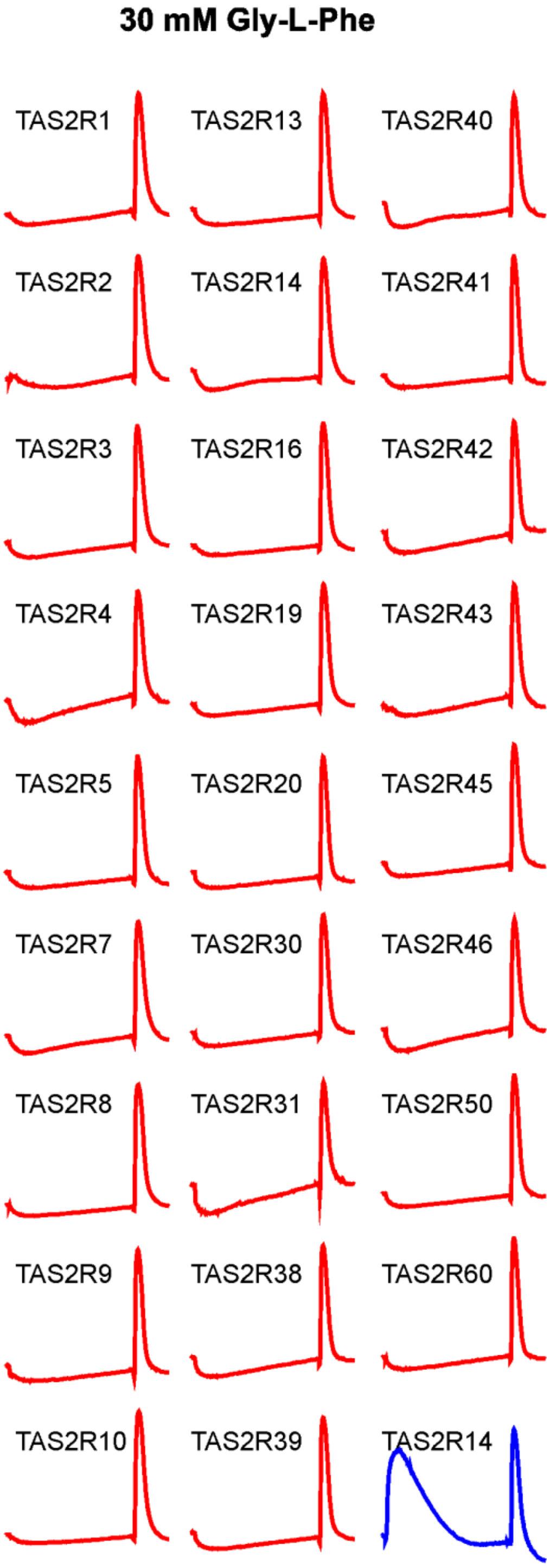

Supplement: Supplementary file 1 [file MNFR-70-e70618-s001.pdf]
